# Combined description (morphology with DNA barcode data) of a new quill mite *Torotrogla paenae* n. sp. (Acariformes: Syringophilidae) parasitising the Kalahari scrub-robin *Cercotrichas paena* (Smith) (Passeriformes: Muscicapidae) in Namibia

**DOI:** 10.1007/s11230-018-9815-z

**Published:** 2018-09-19

**Authors:** Eliza Glowska, Kamila Romanowska, Brian K. Schmidt, Miroslawa Dabert

**Affiliations:** 1 Department of Animal Morphology, Faculty of Biology, Adam Mickiewicz University in Poznan, Umultowska 89, 61-614 Poznan, Poland; 2 Division of Birds, Smithsonian Institution, PO Box 37012, MRC 116, Washington, DC 20013-7012 USA; 3 Molecular Biology Techniques Laboratory, Faculty of Biology, Adam Mickiewicz University in Poznan, Umultowska 89, 61-614 Poznan, Poland

## Abstract

A new quill mite species *Torotrogla paenae* n. sp. (Acariformes: Syringophilidae) parasitising the Kalahari scrub-robin *Cercotrichas paena* (Smith) (Passeriformes: Muscicapidae) in Namibia is described based on the external morphology and DNA barcode data (the mitochondrial cytochrome *c* oxidase subunit 1 sequences, *cox*1). Females of *T. paenae* n. sp. morphologically differ from the most similar species *T. lusciniae* Skoracki, 2004 by the total body length (780–830 *vs* 645–715 µm in *T. lusciniae*) and the presence of hysteronotal shields (*vs* absence), apunctate propodonotal and pygidial shields (*vs* punctate), apunctate coxal fields (*vs* punctate), the fan-like setae *p’* and *p”* of legs III–IV provided with *c.*10 tines (*vs* 14–15) and the length of setae *si* (140–180 *vs* 190–210 µm) and *se* (160–185 *vs* 210–225 µm). The male of *T. paenae* n. sp. morphologically differs from *T. lusciniae* by the lateral branch of peritremes composed of 4 chambers (*vs* 7–8 chambers) and lengths of setae *ve* (45 *vs* 70–75 µm) and *se* (120 *vs* 165 µm).

## Introduction

Quill mites (Acariformes: Syringophilidae) are tiny, permanent bird ectoparasites inhabiting quills of feathers. Syringopilids spend there all their life-cycle and also feed by piercing quill walls with dagger-like chelicerae and suck liquid components of surrounding soft tissue (Casto, 1976; Skoracki, 2011). The family is presently represented by 338 species belonging to 60 genera, which were recorded from 478 bird species from 93 families and 24 orders (Glowska et al., 2015a; Zmudzinski & Skoracki, 2017). The genus *Torotrogla* Kethley, 1970 is widespread globally with proven occurrence in the Palaearctic, Nearctic, Neotropical and Saharo-Arabian zoogeographical regions (Zmudzinski & Skoracki, 2017). Until now, *Torotrogla* has been represented by 18 species associated with several groups of passeriform hosts (35 species and 14 families) (Glowska et al., 2015a, b; Skoracki et al., 2016; Zmudzinski & Skoracki, 2017). Currently, this genus is in the field of molecular research interests, because of the phenotypic plasticity observed in *T. merulae* and *T. rubeculi* (see Glowska et al., 2013) and due to its species being hosts for endosymbiotic bacteria of the genus *Wolbachia* Hertig & Burt 1924 (see Glowska et al., 2015b). All this indicates that basic systematic studies on quill mites, whenever possible, should be extended with DNA data. As it has been shown, molecular tools may be successfully used for syringophilid species descriptions (Glowska et al., 2012a, b), tests of host specificity (Glowska et al., 2016), delimitation of species boundaries (Glowska et al., 2013, 2014) and detecting cryptic species (EG, unpublished data).

In this paper, a new quill mite species *Torotrogla paenae* n. sp. parasitising the Kalahari scrub-robin *Cercotrichas paena* (Smith) (Passeriformes: Muscicapidae) in Namibia is described based on morphological and DNA barcode data. *Cercotrichas paena* is a new host species for the family Syringophilidae and the first record of the genus *Torotrogla* in the Afrotropical realm.

## Materials and methods

*Animal material and morphological analysis*

Mite material used in the study was acquired from the collection of feathers deposited in the Smithsonian Institution, National Museum of Natural History, Department of Vertebrate Zoology, Division of Birds, Washington, DC, USA (USNM) (September 2014). Bird specimen was collected in Namibia, 2009. Drawings were made with an Olympus BH2 microscope with differential interference contrast (DIC) optics and a camera lucida. All measurements are in micrometres. The idiosomal setation follows Grandjean (1939) with modifications adapted for Prostigmata by Kethley (1990). The system of nomenclature for leg chaetotaxy follows that proposed by Grandjean (1944). The application of these chaetotaxy schemes to Syringophilidae was recently provided by Bochkov et al. (2008) with changes by Skoracki (2011). Latin and common names of the bird species follow Clements et al. (2017).

Material depositories and abbreviations: AMU, Adam Mickiewicz University, Poznań, Poland; USNM, Smithsonian Institution, National Museum of Natural History, Washington, DC, USA. The voucher slides and corresponding DNA samples are deposited in the collection of the AMU under the identification numbers as indicated below. The sequences were deposited in the GenBank database under the accession numbers MG948551 (*cox*1) and MG952940 (D1 region of the 28S rRNA gene).

*Molecular data and analysis*

Total genomic DNA was extracted from single specimens using DNeasy Blood & Tissue Kit (Qiagen GmbH, Hilden, Germany) as described by Dabert et al. (2008). We used sequence data for the mitochondrial cytochrome *c* oxidase subunit 1 (*cox*1) gene and the D1 region of the nuclear 28S rRNA gene. *cox*1 was amplified by PCR with degenerate primers Aseq01F (5′-GGA ACR ATA TAY TTT ATT TTT AGA-3′) and Aseq03R (5′-GGA TCT CCW CCT CCW GAT GGA TT-3′) (Glowska et al., 2014). The D1 region of the 28S rRNA gene was amplified using the primer pair 28SF0001 (5′-ACC CVC YNA ATT TAA GCA TAT-3′) (Mironov et al., 2012) and 28SR0450 (5’-TTT GCA ACT TTC CCT CAC GG-3’) (newly designed). PCR amplifications were carried out in 10 µl reaction volumes containing 5 µl of Type-it Microsatellite Kit (Qiagen), 0.5 µM of each primer, and 4 µl of DNA template using a thermocycling profile of one cycle of 5 min at 95°C followed by 35 cycles of 30 s at 95°C, 1 min at 50°C, 1 min at 72°C, with a final step of 5 min at 72°C. After amplification PCR products were two-fold diluted with water, and 5 µl of the sample was analyzed by electrophoresis on a 1.0% agarose gel. Samples containing visible bands were purified with thermosensitive Exonuclease I and FastAP Alkaline Phosphatase (Fermentas, Thermo Scientific, Göteborg, Sweden). The amplicons were sequenced in one direction using PCR forward primers. Sequencing was performed with BigDye Terminator v3.1 on an ABI Prism 3130XL Analyzer (Applied Biosystems, Foster City, CA, USA). Sequence chromatograms were checked for accuracy and edited using FinchTV 1.3.1 (Geospiza, Inc.) and manually aligned in GeneDoc v.2.7.000 (Nicholas & Nicholas, 1997).

**Family Syringophilidae Lavoipierre, 1953**

**Subfamily Syringophilinae Lavoipierre, 1953**

**Genus** ***Torotrogla*** **Kethley, 1970**

***Torotrogla paenae*** **n. sp.**

*Type-host*: *Cercotrichas paena* (Smith) (Passeriformes: Muscicapidae), Kalahari scrub-robin.

*Type-locality*: Omaheke, Aminuis (23°51′01″S, 19°33′42″E), Namibia.

*Type-material*: Holotype female, 2 female and 1 male paratypes (USNM 642341) are deposited in the USNM, 2 female paratypes in the AMU; coll. E. Glowska (10.viii.2009).

*Representative sequences*: GenBank accession numbers for molecular voucher code KR003: MG948551 (*cox*1) and MG952940 (D1 of the 28S rRNA gene).

*ZooBank registration*: To comply with the regulations set out in article 8.5 of the amended 2012 version of the *International Code of Zoological Nomenclature* (ICZN, 2012), details of the new species have been submitted to ZooBank. The Life Science Identifier (LSID) for *Torotrogla paenae* n. sp. is urn:lsid:zoobank.org:act:0F60B0D1-6A08-4C04-8680-6B3BF2C6C295.

*Etymology*: The name is after the specific name of the host.

### Description (Figs. 1–10)

**Figs. 1–5:**
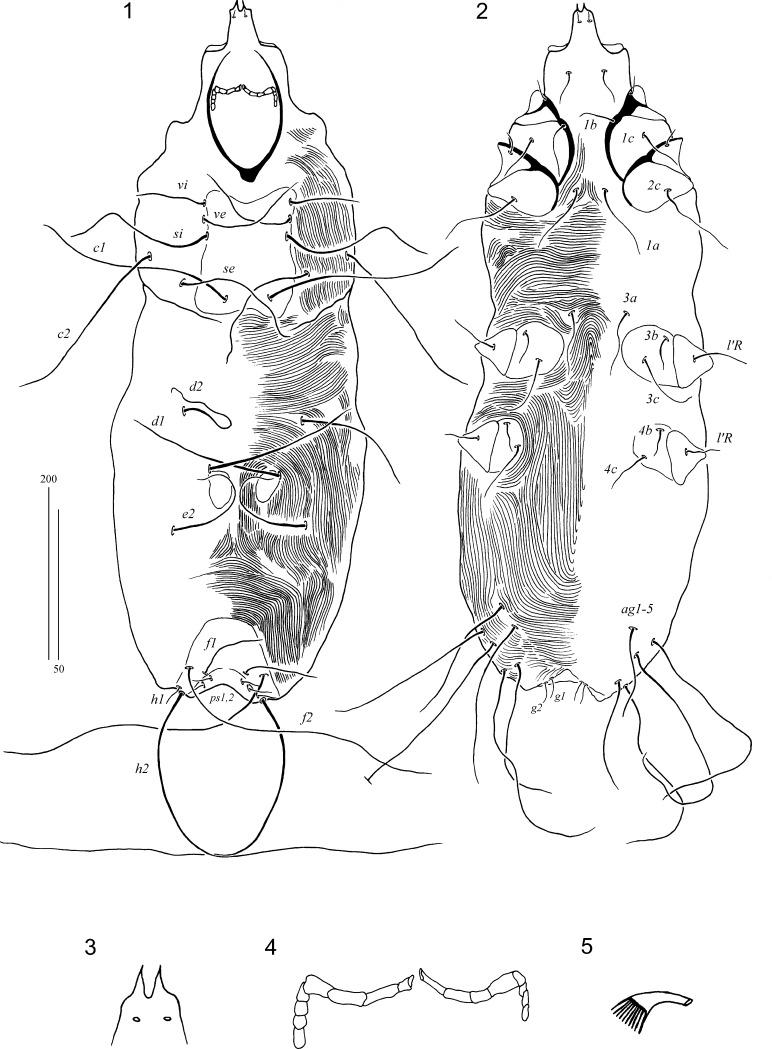
*Torotrogla paenae* n. sp., female: 1, Dorsal view; 2, Ventral view; 3, Hypostomal apex; 4, Peritremes; 5, Fan-like setae *p’* of leg III. *Scale-bars*: 1, 2, 200 µm; 3–5, 50 µm

*Female* [Based on the holotype and 4 paratypes (data in parentheses); Figs. 1–5.] Total body length 800 (780–830 in 4 paratypes). Gnathosoma. Hypostomal apex with pair long, sharp-ended protuberances (Fig. 3). Each medial branch of peritremes with 4 chambers, each lateral branch with 4–6 chambers (Fig. 4). Stylophore constricted posteriorly, 215 (215) long. Idiosoma. Propodonotal shield apunctate, concave on both anterior and posterior margins, bearing bases of setae *vi*, *ve*, *si* and *c1*. Length ratio of setae *vi*:*ve*:*si* 1:1.6–1.7:1.7–2.6. Setae *se* situated anterior to level of setae *c1*. Hysteronotal shields represented by a pair of ovate plates bearing bases of setae *d1*. Length ratio of setae *d2*:*d1*:*e2* 1:1.1–1.4:1–1.5. Pygidial shield well sclerotised and apunctate. Agenital series represented by 5–7 setae on each body side. Legs. All coxal fields apunctate. Setae *tc’* and *tc”* of legs III–IV subequal in length. Fan-like tarsal setae *p’* and *p”* of legs III–IV with *c.*10 tines (Fig. 5). Lengths of setae: *vi* 80 (70); *ve* 130 (105–120); *si* 140 (160–180); *se* 185 (160–185); *c2* 195 (195–215); *c1* 230 (215–245); *d1* 170 (155–185); *d2* 140 (130–150); *e2* 195 (155–200); *f1* 55 (75–90); *f2* 325 (305–370); *h1* 65 (65–70); *h2* 405 (345–375); *ps1* and *ps2* 35 (35–40); *g1* and *g2* 40 (45–55); *3b* 30 (50–60); *3c* 80 (70–90); *4b* 45 (30–60); *4c* 70 (60–105); *tc’III–IV* 70 (65–70); *tc”III–IV* 60 (65–75); *l’RIII* 40 (55–65); *l’RIV* 45 (35–45).

**Figs. 6–10:**
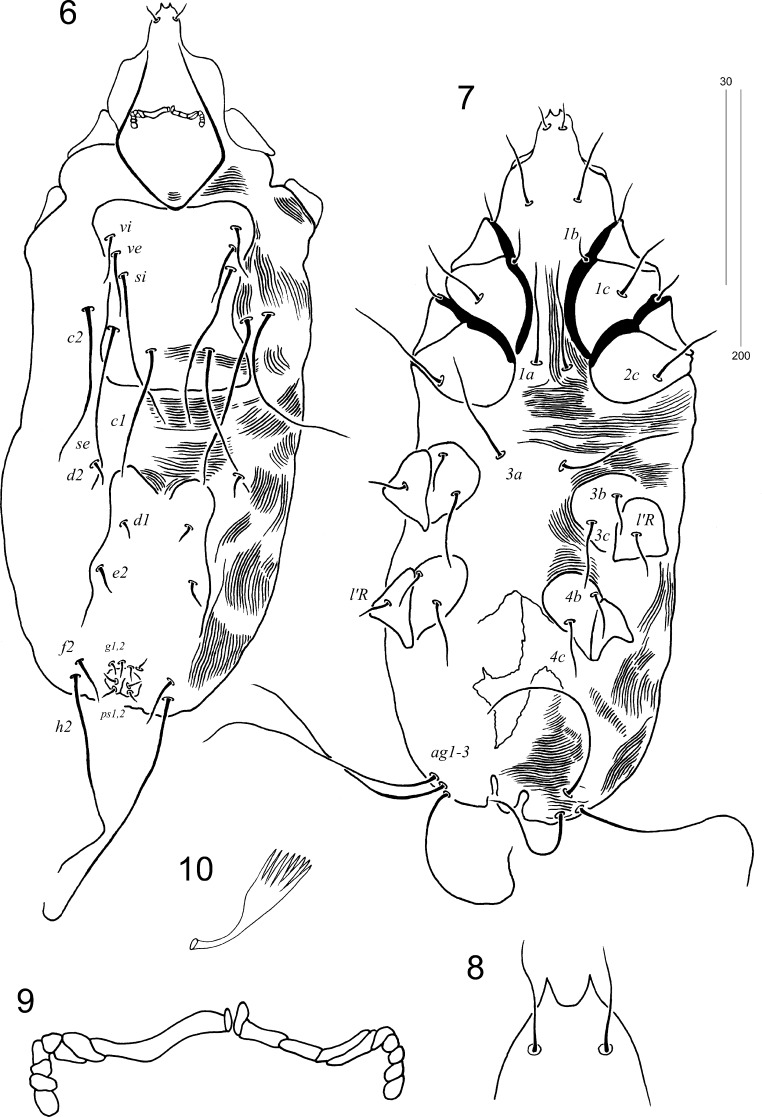
*Torotrogla paenae* n. sp., male: 6, Dorsal view; 7, Ventral view; 8, Hypostomal apex; 9, Peritremes; 10, Fan-like setae *p’* of leg III. *Scale-bars*: 6–7, 200 µm; 8–10, 30 µm

*Male* [Based on 1 paratype; Figs. 6–10.] Total body length 555. Gnathosoma. Hypostomal apex with pair long, sharp-ended protuberances. Each medial branch of peritremes with 4–5 chambers, each lateral branch with 4 chambers (Fig. 9). Stylophore constricted posteriorly, 165 long. Idiosoma. Propodonotal shield apunctate, rectangular in shape, bearing bases of setae *vi*, *ve*, *si* and *c1*. Length ratio of setae *vi*:*ve*:*si* 1:1.1:3. Setae *se* situated anterior to level of setae *c1*. Hysteronotal shield bearing bases of setae *d1*, *e2*, *f2* and *h2*. Length ratio of setae *d2*:*d1*:*e2* 1.5:1:1.5. Setae *h2* 5.8 times longer than *f2*. Agenital series represented by 3 setae on each body side. Setae *g1*,*2* and *ps1*,*2* subequal in length. *Legs.* All coxal fields apunctate. Setae *tc’* and *tc”* of legs III–IV subequal in length. Fan–like setae *p’* and *p”* of legs III–IV with 8 tines. Lengths of setae: *vi* 40; *ve* 45; *si* 120; *se* 120; *c2* 120; *c1* 120; *d1* 20; *d2* 30; *e2* 30; *f2* 35; *h2* 205; *ps1*,*2* 10; *g1*,*2* 10; *3b* 30; *3c* 60; *4b* 45; *4c* 45; *tc’III–IV* 45; *tc”III–IV* 45; *l’RIII* 30; *l’RIV* 20.

DNA barcodes

The *cox*1 sequence data were generated from three females of *Torotrogla paenae* n. sp. All specimens shared the same *cox*1 haplotype (GenBank: MG948551). For one specimen a 305-bp fragment coding for the region D1 of 28S rRNA gene was sequenced as DNA-barcode for nuclear DNA (GenBank: MG952940).

### Remarks

This new species *Torotrogla paenae* n. sp. is morphologically most similar to *T. lusciniae* Skoracki, 2004 described from the common nightingale *Luscinia megarhynchos* Brehm (Muscicapidae) from Italy (Skoracki, 2004). Females of both species have the hypostomal apex provided with a pair of long sharp-ended protuberances, idiosomal setae *f1* and *h1* short and subequal in length, and setae *tc’* and *tc”* of legs III–IV subequal in length. Females of *T. paenae* n. sp. differ from *T. lusciniae* by the following characters: the total body length is 780–830 µm (*vs* 645–715 µm in *T. lusciniae*), the hysteronotal shields are present (*vs* absent), the propodonotal and pygidial shields are apunctate (*vs* punctate), all coxal fields are apunctate (*vs* punctate), the fan-like tarsal setae *p’* and *p”* of legs III–IV are provided with *c.*10 tines (*vs* 14–15) and the length of setae *si* (140–180 *vs* 190–210 µm) and *se* (160–185 *vs* 210–225 µm). Males of *T. paenae* n. sp. differ from *T. lusciniae* in having the lateral branch of peritremes composed of 4 chambers (*vs* 7–8 chambers), and lengths of setae *ve* (45 *vs* 70–75 µm) and *se* (120 *vs* 165 µm).
